# Supplementary material for: Investigation on the strategy of business administration level of enterprises in the new economic environment
Source: PLoS One. 2024 May 9;19(5):e0302931. doi: 10.1371/journal.pone.0302931 (PMC11081260; doi:10.1371/journal.pone.0302931)
Supplement: Attachment — Submitted filename: Response to Reviewers.docx [file pone.0302931.s002.docx]

Review Comments to the Author

Please use the space provided to explain your answers to the questions above. You may also include additional comments for the author, including concerns about dual publication, research ethics, or publication ethics. (Please upload your review as an attachment if it exceeds 20,000 characters)

Reviewer #1: Comment 1: The introduction lacks specific examples or data to support the claims made. Providing concrete examples of the problems faced by Chinese enterprises, as well as tangible instances of successful management strategies, would make the narrative more compelling and convincing.
Answer: According to your suggestion, we add some examples about the problems faced by Chinese enterprises in the introduction, and the specific content is the problems faced by enterprises in China's e-commerce market.
Comment 2: The introduction makes several claims about the problems faced by Chinese enterprises and the necessity of improving management, but there are no references or citations to support these statements. Incorporating relevant literature, studies, or examples from reputable sources would add credibility to the arguments presented.
Answer: According to your suggestion, the introduction adds some content about the success of improving management in China's manufacturing industry, taking Huawei as an example to illustrate the point of view.
Comment 3: Some statements are quite general and lack specificity. For example, the opening sentences broadly state that many scholars have discussed the problems of business management in the new economic environment without specifying the key problems or themes addressed. Be more specific and concise to enhance the relevance of the related work.
Answer: According to your suggestions, the specific content of the discussed issues has been added to the relevant working paragraphs of the article. The main issues include the relationship between corporate innovation and social responsibility, the impact of corporate governance on strategic management accounting disclosure, the expansion of the field of strategic management and corporate risk, and the integration and management process of strategic corporate social responsibility.
Comment 4: The last sentence attempts to introduce a research gap related to enterprise management strategies in the new economic environment. However, the statement lacks specificity and clarity. Clearly articulate what aspects of management strategies have not been explored under the conditions of the new economic environment.
Answer: According to your suggestion, in the last paragraph of the article, we have added aspects that have not been explored in the specific corporate management strategy, including digital transformation, environmental responsibility and globalization strategy.
Comment 5: Some statements in Significance of Improving the Level of Business Management of Enterprises, such as &quot;The economy is developed,&quot; are overly broad and lack specific details or evidence to support the claim. Providing more concrete examples or data to substantiate such statements would strengthen the argument.
Answer: According to your suggestion, in the syllogism of the realization of modern management requirements, the promotion of company innovation and the promotion of economic development, the examples of Ali, Apple and some enterprises' production efficiency are added to strengthen these arguments.
Comment 6: The Significance of Improving the Level of Business Management of Enterprises section mentions the need to &quot;improve the overall quality of the entire enterprise to a certain extent,&quot; but the specific goals or benchmarks for improvement are not clearly outlined. Providing measurable objectives would enhance the clarity of the intended outcomes.
Answer: According to your suggestion, specific improvement targets have been added to this part. This includes improving productivity, reducing costs, improving product quality, strengthening staff, improving customer service, and improving environmental and social responsibility.
Comment 7: The Problems in Business Administration of Chinese Enterprises section suggests that the lack of close MBA training organizations within enterprises contributes to inefficient management training. However, there is no supporting evidence or examples to substantiate this claim. Providing specific instances or studies showcasing the impact of inadequate MBA training on management efficiency would strengthen the argument.
Answer: According to your suggestion, the contents of the Survey Report on the Current Situation of Chinese Enterprise Management have been added to this part to strengthen the adequacy of the argument that insufficient MBA training leads to inefficient management training.
Comment 8: The conclusion mentions that as enterprises adapt to the new environment, the business management level will become more comprehensive. However, it lacks specific insights into how this improvement will occur or what specific aspects of management will see enhancement. Providing more details on the expected improvements would strengthen the conclusion.

Answer: According to your suggestion, specific expected contents have been added in this part to strengthen the conclusion, including technology integration and innovation, human resource development, strategic planning and execution, corporate culture and leadership, financial management and risk control, customer relationship management, continuous improvement and learning, etc.

Reviewer #2: During the review I have found some issues to inform the author(s). Those are given below:

About grammatical improvements;
Fragmented Sentences:
Original: &quot;Under the conditions of the new economic environment, in order to achieve long-term development, enterprises must continuously improve their capabilities.&quot;
Suggested: &quot;In the face of the new economic environment, enterprises must continuously enhance their capabilities to achieve long-term development.&quot;
Answer: This sentence has been revised according to your suggestion.
Redundancy:
Original: &quot;Under the current market situation, business management is a kind of knowledge based on economic management and legal accounting.&quot;
Suggested: &quot;In the current market scenario, business management relies on economic principles and legal accounting.&quot;
Answer: This sentence has been revised according to your suggestion.
Repetition:
Original: &quot;In view of the current market situation, the article analyzed the enterprise&rsquo;s system reform and production planning, and gave corresponding countermeasures.&quot;
Suggested: &quot;Considering the current market situation, the article analyzed enterprise system reform and production planning, proposing corresponding countermeasures.&quot;
Answer: This sentence has been revised according to your suggestion.
Ambiguity:
Original: &quot;By comparing with the traditional management model in the complexity of enterprise management process, enterprise management efficiency, management level score, and enterprise quarterly profit...&quot;
Suggested: &quot;Comparing with the traditional management model in terms of the complexity of enterprise management processes, efficiency, management level score, and quarterly profit...&quot;
Answer: This sentence has been revised according to your suggestion.
Awkward Phrasing:
Original: &quot;It has also been explained that under the new economy, the management level of enterprises needs to be improved in order to enable enterprises to achieve long-term development.&quot;
Suggested: &quot;Furthermore, it is elucidated that, in the new economy, enhancing the management level is essential for enabling enterprises to attain long-term development.&quot;
Answer: This sentence has been revised according to your suggestion.
Numbers and Units:
Original: &quot;It has been found that the management model under the new economic environment has reduced the complexity of enterprise process by 0.17.&quot;
Suggested: &quot;Findings reveal that the management model in the new economic environment has reduced the complexity of the enterprise process by 0.17 points.&quot;
Answer: This sentence has been revised according to your suggestion.
Consistency:
Ensure consistent use of either singular or plural forms, e.g., &quot;enterprise&#39;s system reform&quot; versus &quot;enterprises needed.&quot;
Answer: According to your suggestion, change "enterprise system reform" to "enterprises system reform".
Clarity:
Consider restructuring sentences for clearer expression and avoiding overly complex sentence structures.
Answer: According to your suggestion, when revising the content of the article, the modified part reduces the complex sentence structure.
Introduction:
the introduction appropriately sets the stage for discussing the challenges and opportunities faced by enterprises in the evolving economic landscape and lays the groundwork for the subsequent detailed analysis.
Answer: According to your suggestion, a discussion on the challenges and opportunities facing enterprises in the changing economic environment has been added in the last paragraph of the introduction to lay the foundation for the argument and in-depth analysis of the full text.
Literature/Related works:
Authors need to mention more rigorously the absolute and relative gap and the importance to bridge the it.
Answer: According to your suggestion, the last part of the relevant work has been revised, and the discussion on the gap in enterprise management strategy has been added, and the importance of closing this gap has been reflected.
The use of the phrase &quot;under the new economic environment&quot; is repeated multiple times. Consider varying the language for better flow and clarity.

Answer: According to your suggestion, replace the part of "under the new economic environment" with other words.

The transition between different studies and their findings could be smoother. Consider using transition sentences to link the various research topics more cohesively.
Answer: According to your suggestions, we have modified the narration among various research topics in the literature review and added some excessive words and sentences appropriately to make the article more smooth.
In the summary of research findings, some statements are general and could benefit from more specific details. For example, when discussing Honggowati S&#39;s research, provide more information about the findings beyond general statements like &quot;management&rsquo;s shareholding had a negative impact on the level of strategic management disclosure.&quot;
Answer: According to your suggestion, the description in the Honggowati S study has been modified to reflect the results of the study with more specific data.
Ensure that the citation style is consistent throughout the review. For instance, some citations are presented with the author&#39;s name followed by the initial, while others include the full name.
Answer: According to your suggestion, the format of the quotation should be changed to the author's name and initial.
While the review mentions various studies, it does not delve deeply into the methodologies or specific findings of each study. Providing a bit more detail on the methodologies and key findings would enhance the depth of the review.
Answer: According to your suggestion, the contents of some literatures have been changed, and discussions on specific methods and research have been added.
The statement, &quot;In the aspect of enterprise business management, scholars have conducted many discussions, but no research has been done on enterprise management strategies,&quot; lacks specificity. It would be beneficial to clearly identify the specific gap in the literature regarding enterprise management strategies.
Answer: According to your suggestion, a discussion on the differences between different studies has been added at the end of the relevant work.
Methodology:
Author may use some established methodology for this type of study. in this study, the method is not clear enough. kindly mention and give some reasoning why and why not using different methods.

Answer: Based on your suggestions, a clear approach has been added to the HR optimization section, which includes strengthening interdisciplinary teamwork, training managers in digital skills, establishing flexible management systems, promoting continuous innovation and iteration, and collaborating with external experts and consultants.

Reviewer #3: First and foremost, I want to commend you on your diligent work in exploring a research topic that holds significant novelty and relevance in the current economic landscape. Your investigation into the challenges and opportunities facing enterprises in the new economic environment is both timely and insightful, and it reflects a commendable dedication to advancing our understanding of contemporary business management practices.
While your research undoubtedly contributes valuable insights to the field, I believe there are several areas where further improvements can be made to enhance the quality and impact of your paper. Here are some suggestions for refinement:
1. Enhanced Structure and Clarity:
&bull; Consider revising the structure of your paper to provide a clearer roadmap for readers, guiding them through the literature review, methodology, results, and discussion sections in a logical sequence.
&bull; Ensure that each section is well-defined and articulated, with clear transitions between ideas and arguments.

Answer: According to your suggestion, an introduction of the structure of this paper and a structure diagram are added at the end of the introduction. In the introduction, the writing idea of this paper is clearly introduced, and the transition part is added. Picture:

2. Deeper Literature Review:
&bull; Expand your literature review to encompass a broader range of scholarly works and perspectives relevant to your research topic.
&bull; Provide more detailed analysis and critique of existing literature, highlighting gaps, contradictions, and areas for further investigation.

Answer: According to your suggestion, two related literatures on the impact of digital transformation on enterprise management have been added, and the descriptions of existing literatures in the literature review have been modified.
3. Methodological Rigor:
&bull; Strengthen the methodological approach employed in your research, ensuring that data collection and analysis procedures are well-defined and rigorously executed.
&bull; Consider incorporating additional research methods or approaches to enrich the depth and robustness of your findings.

Answer: According to your suggestion, section 3.5 is added to the paper, which discusses the method of data collection in the questionnaire survey, so as to make the collected data more scientific and rich, and make the results complete. In addition, the part of data collection was modified in the discussion before the experiment to make the process more rigorous.
4. Evidence-Based Analysis:
&bull; Ground your analysis and discussion in empirical evidence, drawing on real-world examples, case studies, or statistical data to support your arguments and conclusions.
&bull; Clearly present your findings in a systematic and transparent manner, allowing readers to assess the validity and reliability of your research outcomes.

Answer: According to your suggestion, I revised the discussion of the results of the comparison of different management modes in this paper to make the research results more intuitive, and added examples about restaurants and retail stores to support the argument and conclusion.
5. Concluding Insights and Implications:
&bull; Offer insightful reflections on the implications of your findings for theory, practice, and future research directions.
&bull; Highlight the practical implications of your research for business leaders, policymakers, and other stakeholders, emphasizing actionable recommendations for improving enterprise management in the new economic environment.

Answer: According to your suggestion, the statement in the conclusion part of the article has been revised to add the part of practical significance for business leaders, policy makers and other stakeholders, emphasizing feasible suggestions for improving corporate management in the new economic environment.
By incorporating these suggestions into your paper, you can further elevate its quality and significance, ultimately making a more substantial contribution to the academic discourse on enterprise management and the evolving economic landscape.
